# Highly sensitive and repeatable DNA‐SERS detection system using silver nanowires‐glass fiber filter substrate

**DOI:** 10.1002/ansa.202000096

**Published:** 2020-10-16

**Authors:** Ayoung Woo, Kyongmook Lim, Baek Hwan Cho, Ho Sang Jung, Min‐Young Lee

**Affiliations:** ^1^ Department of Medical Device Management and Research, Samsung Advanced Institute for Health Sciences & Technology Sungkyunkwan University Seoul Republic of Korea; ^2^ Smart Healthcare Research Institute, Biomedical Engineering Research Center Samsung Medical Center Seoul Republic of Korea; ^3^ Advanced Nano‐Surface Department Korea Institute of Materials Science (KIMS) Changwon Republic of Korea

**Keywords:** glass fiber filter, loop‐mediated isothermal amplification, on‐site diagnostics, silver nanowire, surface‐enhanced Raman spectroscopy

## Abstract

This paper describes a new simple DNA detection method based on surface‐enhanced Raman scattering (SERS) technology using a silver nanowire stacked‐glass fiber filter substrate. In this system, DNA‐intercalating dye (EVAGreen) and reference dye (ROX) are used together to improve the repeatability and reliability of the SERS signals. We found that the SERS signal of EVAGreen was reduced by intercalation into DNA amplicons of a polymerase chain reaction on the silver nanowire stacked‐glass fiber filter substrate, whereas that of ROX stayed constant. The DNA amplicons could be quantified by correcting the EVAGreen‐specific SERS signal intensity with the ROX‐specific SERS signal intensity. Multivariate analysis by partial least square methods was also successfully performed. And we further applied it to loop‐mediated isothermal amplification with potential use for on‐site diagnostics. The sensitivities of the DNA‐SERS detection showed about 100 times higher than those of conventional fluorescence‐based detection methods. The DNA‐SERS detection method can be applied to various isothermal amplification methods, which is expected to realize on‐site molecular diagnostics with high sensitivity, repeatability, simplicity, affordability, and convenience.

## 1 INTRODUCTION

Nucleic acid biosensors have tremendous potential in many fields especially in clinical, environmental, and food analysis.^1^ With growing demands for on‐site molecular diagnostics, there are still needs for the technological improvements in terms of simplicity, convenience, rapidity, affordability, and sensitivity of detection.^2^ Fluorescence, as a pioneering tool, has been used in most nucleic acid analysis platforms such as polymerase chain reaction (PCR).^3^, ^4^ However, fluorescence‐based analysis has intrinsic shortcomings such as high susceptibility to photobleaching, requiring long DNA amplification time for detectable levels, nonspecific signals, broad emission spectrum limiting their potential for high‐level multiplex, and requiring multiple excitation laser lines for multiplex detection.^5^, ^6^

Surface‐enhanced Raman spectroscopy (SERS) is rapidly emerging as a promising on‐site diagnostic tool due to high sensitivity, multiplex detection using portable equipment with single excitation source and detector, and so on.^7^, ^8^ SERS enhances Raman scattering of molecules adsorbed onto regions of highly enhanced electromagnetic fields formed on the enhancing substrates. It has become apparent that the presence of nanogaps or nanoparticle junctions on the substrates (usually called a hotspot) is responsible for a large part of the enhancement via localized collective oscillations of free surface elctrons.^9^ Recently, several studies on DNA detection systems using SERS technology have been reported. White et al demonstrated PCR‐SERS detection systems using hydrolysis probe, in which hydrolyzed dye should be separated by lateral flow^10^ or dialysis tube^11^ for SERS detection. Zhou et al demonstrated PCR‐SERS detection system using a silver nanoparticle solution with DNA intercalating dye,^12^ but it may be difficult to control the arrangement of metal nanoparticles in a solution containing various substances such as nucleotide, primers, enzymes, ions, and so on, which limits the repeatability of the SERS signal.

On the other hand, nucleic acid isothermal amplification technologies have attracted attention as alternative methods of thermocycling‐based PCR for on‐site molecular diagnostics.^13^ The isothermal amplification enables rapid amplification and simplifies/miniaturizes the equipment. Among them, loop‐mediated isothermal amplification (LAMP) is a promising method based on its specificity, simplicity, rapidity, low cost, and resistance to inhibitors of complex clinical samples.^14^ For on‐site diagnostic application, LAMP can be simply analyzed by visual inspection of the color changes caused by pH‐sensitive dyes, the fluorescence changes caused by DNA intercalating dyes, or turbidity changes caused by white precipitation of magnesium pyrophosphate.^15^, ^16^, ^17^, ^18^ However, the visual inspection of the changes is difficult at very low levels of target gene. Furthermore, DNA intercalating dye needs to be used at a high concentration for fluorescence change (∼1000‐fold higher than that for quantitative real‐time PCR), which interferes with amplification due to inhibitory effect,^15^ and may cause fluorescence changes with nonspecific binding to primers, resulting in false positive results.^15^

Here, we report a new simple DNA‐SERS detection system using a silver nanowires stacked‐glass fiber filter (AgNWs‐GFF) substrate. We found that SERS signal of EVAGreen was reduced on the substrate as it intercalated into DNA amplicon. This is because the dye intercalated into DNA is difficult to be adsorbed to the surfaces or nanogaps of AgNWs due to large size and electrostatic repulsion. We also introduced ROX as an internal standard dye to ensure the repeatability and reliability of the SERS signal. This makes it possible to detect the presence of the DNA amplicon by observing the relative intensities of the SERS vibrational bands. In addition, it is possible to quantify it by comparing the SERS signal intensities of EVAGreen and ROX even without a calibration curve. Because the EVAGreen and ROX are used in this system at a concentration level used for PCR, it does not interfere with nucleic acid amplification. In this article, we show PCR‐SERS detection and LAMP‐SERS detection using a portable Raman spectroscopy with a single excitation laser.

## 2 MATERIALS AND METHODS

### 2.1 Materials

AgNWs (average diameter of about 40 nm and an average length of about 8 µm, 0.3 wt%) coated with polyvinylpyrrolidone (PVP) was purchased from Nanopyxis Co, Ltd (Seoul, Republic of Korea) (Figure S1). Glass fiber filter with pore size of about 0.7 µm (GFF, model no. 1825‐047) was purchased from Whatman (Maidstone, UK). Complimentary DNA (cDNA) synthesis kit was purchased from Thermo Fisher Scientific (Waltham, MA, USA). Primers and templates were synthesized by Bioneer (Daejeon, Republic of Korea). Real‐time PCR master mix was purchased from Toyobo (Osaka, Japan) and PCR master mix without any dye was purchased from Takara (Shiga, Japan). WarmStart LAMP kit was purchased from New England Biolabs (Ipswich, MA, USA). EVAGreen (20×, 25 µM) was purchased from Biotium (Fremont, CA, USA) and ROX (50×, 25 µM) was purchased from Bio‐Rad (Hercules, CA, USA).

### 2.2 Fabrication of AgNWs‐GFF substrate

The AgNWs‐GFF substrate was fabricated by vacuum filtration of a dispersion solution of 0.3 wt% AgNWs onto a GFF as previously reported.^18^ After the filtration, the AgNWs‐GFF substrate was washed with distilled water several times and dried on a hot plate at 170°C for 5 min. The AgNWs‐GFF substrate was cut into 40 mm × 40 mm and used for SERS analysis.

### 2.3 SERS analysis of EVAGreen and ROX dyes

Each of three microliter of EVAGreen (1×), ROX (1×), or their mixture (EVAGreen:ROX = 2:1, 1:1, 0.75:1, 0.5:1, 0.25:1, and 0.125:1) was dropped onto the substrate, and SERS signals were obtained after drying for 5 min on a hotplate at 60°C using a portable Raman spectrometer (Nanoscope Systems, Inc, Daejeon, Republic of Korea) following measurement conditions: exposure time, 5 s; laser power, 5 mW; and laser wavelength, 633 nm. All spectra were collected three times at random points from the three sets of samples prepared under the same conditions (n = 3) and averaged for plotting. The portable Raman spectrometer provides Raman intensity values every 3.504 cm^−1^ between 100 and 3000 cm^−1^. We used the Raman intensity values at specific peaks for quantification.

### 2.4 Polymerase chain reaction

Influenza A virus RNA and coronavirus 229E RNA were purchased from the Korea Bank for Pathogenic Viruses (KBPV, Seoul, Republic of Korea) and their cDNA was synthesized using the cDNA synthesis kit. Forward and reverse primers for influenza A virus were synthesized as 5′‐GTCCAACCCTAAGTCCAA‐3′ and 5′‐GCCACAAAACACAACAATAC‐3′, respectively. Real‐time PCR was performed using the real‐time PCR master mix by QuantStudio 6 Flex real‐time PCR system (Life Technologies, Carlsbad, CA, USA) coupled with QuantStudio real‐time PCR software (v.1.1; Life Technologies). The real‐time PCR amplification conditions were as follows: 95°C predenaturation for 1 min, followed by 35 cycles of a denaturation at 95°C for 15 s, and extension at 60°C for 45 s. PCR was also performed using the PCR master mix by Applied Biosystems Veriti 96‐Well Thermal Cycler (Thermo Fisher Scientific). The amplification conditions were as follows: 95°C predenaturation for 1 min, followed by 35 cycles of a denaturation at 95°C for 15 s, and extension at 60°C for 45 s. The amplified PCR products were identified by electrophoresis using a 2% agarose gel.

### 2.5 Loop‐mediated isothermal amplification

*Chlamydia pneumoniae ompA* gene was synthesized and used as a target template for LAMP. *Streptococcus pneumoniae ply* gene was synthesized and used as a nontarget template. LAMP primers for the *OmpA* gene were designed by Primer Explorer (v.4.0) and were synthesized as F3, 5′‐AATGAACTACCAAACGTTTCT‐3′; B3, 5′‐CTTGGGTTTGTTTACAGAGAA‐3′; FIP, 5′‐CATTCCCATAAAGCTCCACGAGATGGAGTTGTTGAACTCTACAC‐3′; and BIP, 5′‐CGGTTGTGCAACTTTGGGAGGTTACAGATCACATTAAGTTCTTCA‐3′. LAMP was performed using the WarmStart LAMP kit according to its protocol with slight modification. LAMP was conducted under isothermal condition of 65°C for 30 min. The LAMP products were analyzed by gel electrophoresis using a 2% agarose gel. Also, the LAMP products with EVAGreen (3×) were confirmed by visual inspection under UV illumination.

### 2.6 SERS analysis of DNA amplicon

The AgNWs‐GFF substrate was cut into 40 mm × 40 mm. Three microliter of PCR products with EVAGreen (1×) and ROX (1×) or LAMP products with EVAGreen (3×) and ROX (1×) was dropped onto the substrate, and SERS signals were obtained after drying for 5 min on the hotplate at 60°C using a portable Raman spectrometer (Nanoscope Systems, Inc) following measurement conditions: exposure time, 5 s; laser power, 5 mW; and laser wavelength, 633 nm. All spectra were collected three times at random points from the three sets of samples prepared under the same conditions (n = 3) and averaged for plotting.

### 2.7 Data analysis

Partial least square (PLS) regression and PLS discriminant analysis were carried out by threefold cross‐validation in python 3.6 environment using EVAGreen specific peaks (615, 870, 1235, 1368, 1490, and 1529 cm^−1^) and ROX specific peaks (692, 755, 840, 1224, 1452, and 1501 cm^−1^).

## 3 RESULTS AND DISCUSSION

### 3.1 Fabrication of AgNWs‐GFF substrate

Noble metal particle solution‐based nanoscale building blocks are common means of obtaining strong SERS signals due to their ease of synthesis.^19^ However, it is difficult to achieve high repeatability of SERS measurements due to irregular particle aggregation that can be occurred during SERS application. Precisely fabricated nanostructures can be manufactured uniformly; however, they typically require expensive equipment and extended production times.

**FIGURE 1:**
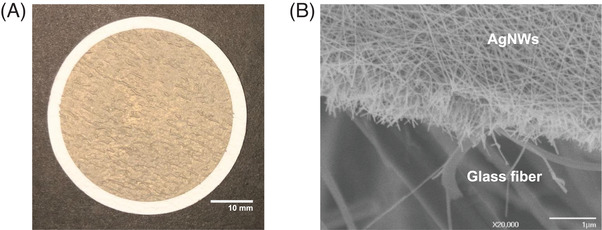
Characterization of AgNWs‐GFF substrate: (A) photograph and (B) field emission scanning electron microscope image of AgNWs‐GFF substrate

In the present study, we fabricated AgNWs‐GFF substrate by vacuum filtration of AgNWs (average diameter of about 40 nm and an average length of about 8 µm; Figure S1) coated with a negatively charged PVP onto porous GFF (Figure 1A). Because the average pore size of GFF is 700 nm, AgNWs with an average length of 8 µm cannot pass through the pore and can be densely stacked on the GFF, forming networks and nanopore structures. The porosity and large surface area of the AgNWs‐GFF substrate can enable high absorption of water and analytes. The field emission scanning electron microscopy image (Figure 1B) showed that AgNWs were deposited in a three‐dimensional matrix on GFF with high density. The regions of intersected AgNWs can act as hotspots and the three‐dimensional stacked AgNWs can greatly increase the SERS effect by allowing for additional hotspots.^18^ Although the hotspots are formed randomly at the nanoscale, their density is >355 points/µm^2^ and shows uniform coverage across the entire GFF.^20^ Considering a Raman laser spot area of 2.22 µm^2^ used for SERS detection, the inclusion of >700 hotspots in a single laser spot can overcome the nanoscale randomness of the SERS substrate.^20^

### 3.2 DNA‐SERS detection strategy using AgNWs‐GFF substrate

**FIGURE 2:**
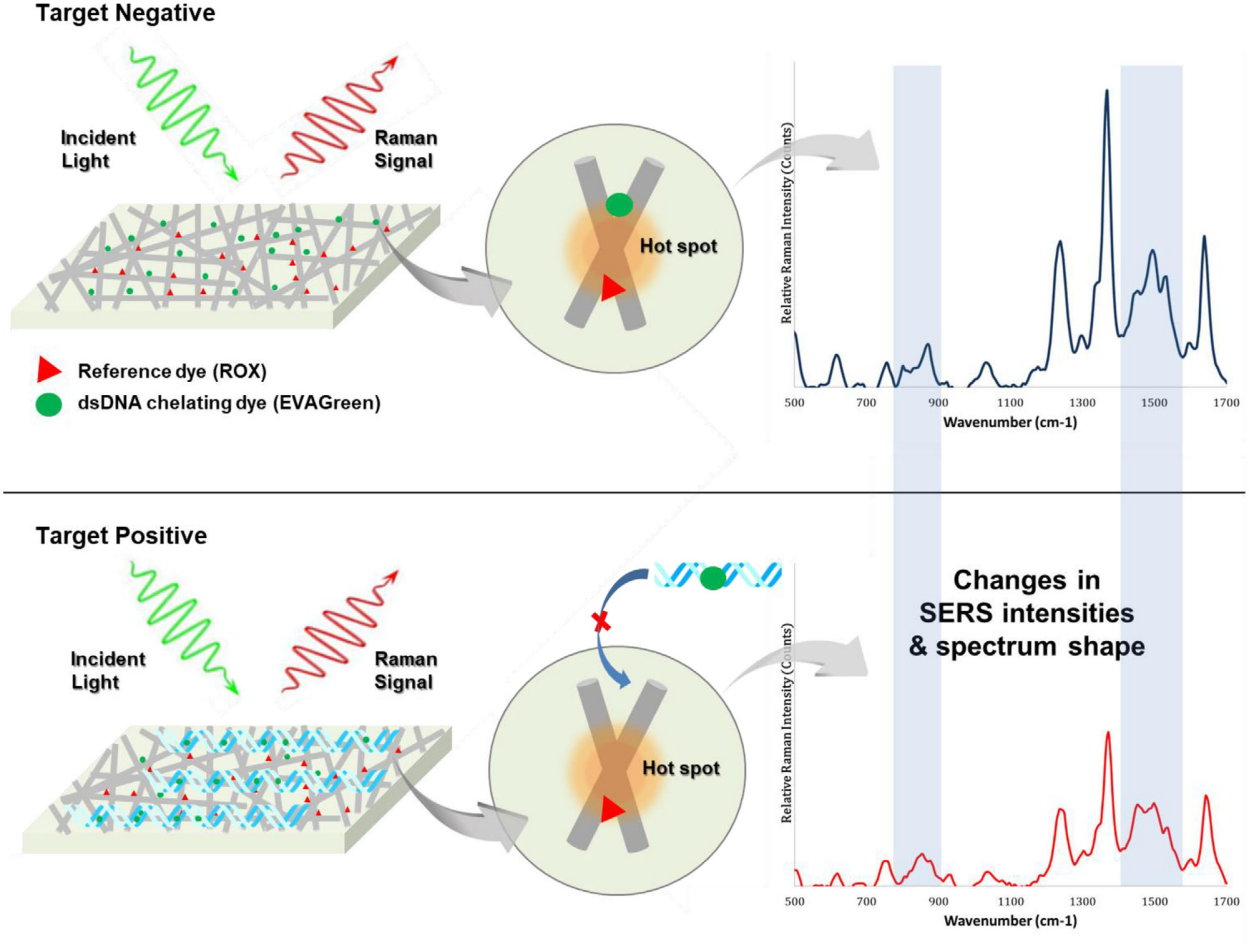
Schematic illustration of DNA‐SERS detection system using AgNWs‐GFF substrate

We found that, on the AgNWs‐GFF substrate, small molecules such as amino acids (tryptophan, tyrosine, and phenylalanine) showed significant SERS signals, whereas large molecules such as DNA (synthetic 260 bp, genomic about 5 000 000 bp) and proteins (streptavidin; about 53 kDa, bovine serum albumin; about 66 kDa, digoxigenin antibody; about 150 kDa) did not show significant signals even at a high concentration of 1 mg/mL. It might be due to that the small molecules are closely adsorbed to the hotspots of the AgNWs‐GFF substrate, whereas the DNA and proteins are difficult to be adsorbed to the surfaces and nanogaps of the AgNWs due to large size and/or electrostatic repulsion. From these phenomenon, we expected that in the absence of dsDNA, the SERS signal of DNA intercalating dye (EVAGreen) is enhanced on the AgNWs‐GFF substrate, whereas in the presence of dsDNA with large size and negative charge, the SERS signal of EVAGreen is decreased due to intercalation into the dsDNA. Although SERS technology allows high‐sensitivity detection, variations in SERS intensity can be generated by nonuniformity of hotspot formation, errors in sampling and laser focusing steps, background signals, and so on. In order to improve the accuracy of SERS analysis, internal standards have been used to normalize SERS signal variations.^21^, ^22^, ^23^ In this work, we introduced ROX as a reference dye that does not interact with dsDNA to increase the repeatability and reliability of the SERS signal for the dsDNA detection. Therefore, we hypothesized that in the absence of a target gene, the SERS signals derived from EVAGreen and ROX on the AgNWs‐GFF SERS substrate would be enhanced in combination, whereas in the presence of DNA amplified from a target gene, the SERS signal derived from EVAGreen would be reduced relative to that of ROX (Figure 2).

### 3.3 SERS signals of EVAGreen and ROX on AgNWs‐GFF substrate

**FIGURE 3:**
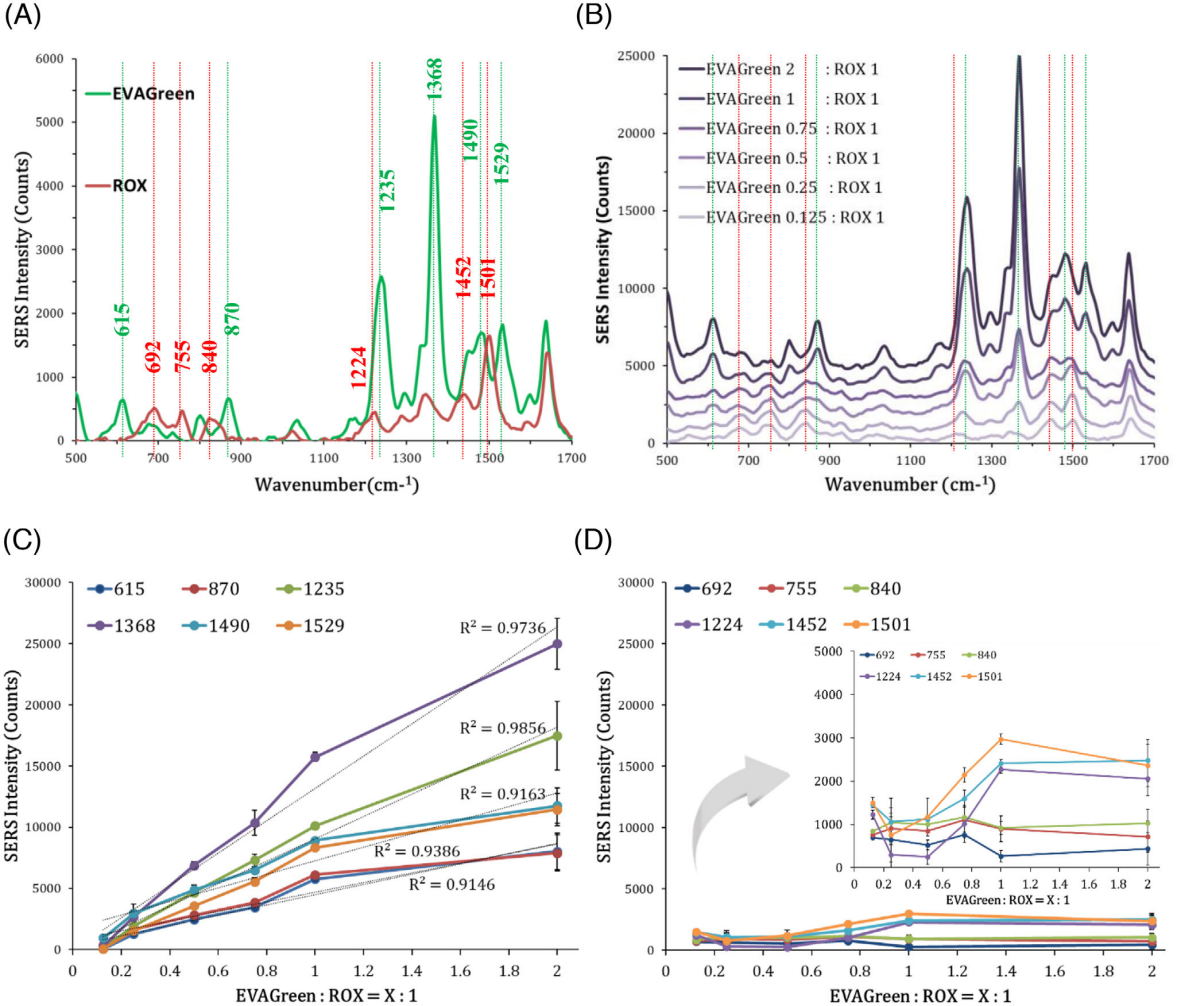
(A) SERS spectra of EVAGreen and ROX. (B) SERS spectra of mixtures of EVAGreen and ROX according to ratios. Green lines are EVAGreen‐specific peaks and red line are ROX‐specific peaks. Plot of SERS intensity of (C) EVAGreen‐specific peaks and (D) ROX‐specific peaks according to the ratios of EVAGreen to ROX

Each EVAGreen (1×) and ROX (1×) solution was dropped onto the AgNWs‐GFF substrate, and SERS spectra were obtained using a portable Raman analyzer with laser wavelength of 633 nm and laser spot area of 2.22 µm^2^ (Figure 3A). The SERS spectra normally contain background scattering, so baseline correction (background subtraction) is a crucial step in the preprocessing of Raman spectra.^24^, ^25^ In this work, baseline correction of SERS spectra was carried out by using NSRamanID software that uses asymmetric least square method,^24^ provided by the Raman instrument. The EVAGreen and ROX dyes can be closely adsorbed to the surface of PVP‐coated AgNWs after drying, and the negatively charged PVP can interact with the dyes through the charge interaction with the positive residues of the dyes and Van der Waals interactions. EVAGreen showed specific peaks at 615, 870, 1235, 1368, 1490, and 1529 cm^−1^, whereas ROX showed specific peaks at 692, 755, 840, 1224, 1452, and 1501 cm^−1^. Figure 3B shows the SERS spectra of mixtures of EVAGreen and ROX at ratios of 2:1, 1:1, 0.75:1, 0.5:1, 0.25:1, and 0.125:1. As the ratio of EVAGreen to ROX decreased, the intensity of the specific EVAGreen peaks decreased gradually and the ROX‐specific peaks became more evident in the spectra of two components. Plotting of the SERS signal intensities of the mixtures according to the ratios showed that the EVAGreen‐specific peaks displayed a high linear correlation coefficient (>0.9) at all peaks (Figure 3C). The ROX‐specific peaks at 692, 755, and 840 cm^−1^ showed almost constant SERS signal intensities regardless of the ratio with EVAGreen. On the other hand, the ROX‐specific peaks at 1224, 1452, and 1501 cm^−1^ exhibited slightly increased intensities as the ratio of EVAGreen to ROX increased, which is because that EVAGreen‐specific peak (1235, 1368, 1490, and 1529 cm^−1^) areas are located close to them (Figure 3D), but it is negligible compared to the changes of the EVAGreen signal intensities. It was predicted that when a mixture of EVAGreen and ROX was added to the nucleic acid amplification products, EVAGreen would be inserted into the dsDNA, reducing the signals, whereas ROX would not be inserted into the dsDNA, thus maintaining a constant signal.

### 3.4 PCR‐SERS detection

**FIGURE 4:**
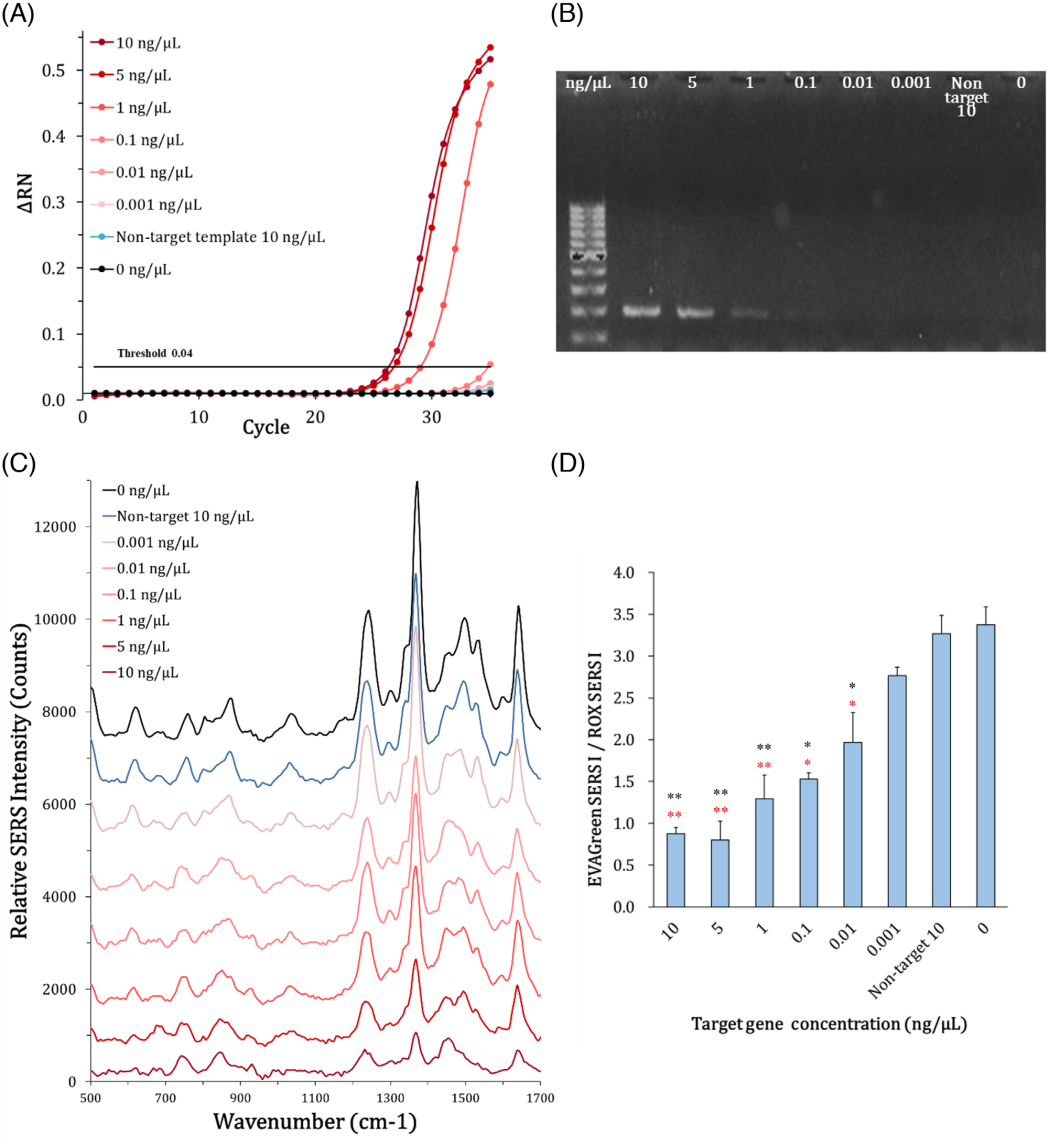
PCR analysis. (A) Real‐time PCR and (B) agarose gel electrophoresis of PCR products. (C) SERS spectra of PCR products with EVAGreen and ROX on the AgNWs‐GFF substrate. (D) Bar graph of the EVAGreen SERS intensity at 870 cm^–1^/ROX SERS intensity at 840 cm^–1^ according to target template concentration (**P < .005 and *P < .05; black * is against blank and red * is against nontarget template)

Real‐time PCR and gel electrophoresis were performed as comparisons with PCR‐SERS detection. PCR was performed using a target gene at a final concentration range of 0.001 to 10 ng/µL. Blank (without template) and nontarget template 10 ng/µL were used as negative controls. The real‐time PCR analysis confirmed that the target gene was detected down to a concentration of 1 ng/µL (Figure 4A), which was the same as the results of gel electrophoresis (Figure 4B). The amplified DNA of 196 bp (∼70 nm length) showed a band around 200 bp on the gel indicating proper amplification of the target gene. PCR results of the blank and nontarget template showed no amplification.

The PCR products with EVAGreen (1×) and ROX (1×) were dropped onto the AgNWs‐GFF substrate, and SERS spectra were obtained using the portable Raman equipment. The baseline correction of SERS spectra was carried out by using the NSRamanID software (Figure 4C). The SERS signals of EVAGreen and ROX were similarly enhanced in the blank and nontarget template 10 ng/µL. No other significant signals were observed in the spectra other than EVAGreen‐ and ROX‐specific peaks. In the presence of the target gene, the EVAGreen‐specific SERS signals at 615, 870, 1235, 1368, 1490, and 1529 cm^−1^ decreased gradually according to the concentration of the target gene, whereas ROX‐specific SERS signals at 692, 755, 840, and 1452 cm^−1^ showed no distinguishable changes. As the concentration of the target gene increased, the ROX‐specific peaks became more evident in the spectra of the PCR products with EVAGreen and ROX. These suggest that EVAGreen intercalated into DNA amplicon of PCR is difficult to be adsorbed onto the surfaces and nanogaps of AgNWs on the substrate due to the large size and/or negative charge of the DNA amplicon, thereby resulting in reduced EVAGreen‐specific SERS signals. In particular, in the SERS spectral ranges of 800‐900 and 1450‐1550 cm^−1^, the groups containing the target gene from 0.01 to 10 ng/µL were highly distinguished from those of the blank and nontarget template based on the relative intensities of the SERS vibrational bands without SERS intensity‐based calculations. This is because the ROX peaks were more evident as the EVAGreen peaks decreased. The low concentrations of the target gene of 0.1 and 0.01 ng/µL, which were under threshold in the real‐time PCR and undetectable by the gel electrophoresis, were distinguishable from the blank and nontarget template. In the observation of SERS spectra, the lowest concentration of the target gene of 0.001 ng/µL was not distinguishable from the nontarget template.

To quantify the DNA amplicon from the SERS spectra, the EVAGreen‐specific SERS signal intensity at 870 cm^−1^ (EVAGreen SERS I) was corrected with the ROX‐specific SERS signal intensity at 840 cm^−1^ (ROX SERS I). As shown in Figure 4D, it was confirmed that the EVAGreen SERS I/ROX SERS I value decreased as the concentration of target gene increased. The EVAGreen SERS I/ROX SERS I values were 3.4, 2.8, 2.0, 1.5, 1.3, 0.80, 0.87, and 3.3 for the target templates of 0,0.001, 0.01, 0.1, 1, 5, 10 ng/µL and nontarget template 10 ng/µL, respectively. The amplification appears to be saturated above 5 ng/µL of the target gene. Even at the low target gene concentrations of 0.001, 0.01 and 0.1 ng/µL, the EVAGreen SERS I/ROX SERS I value was about 1.2, 1.7, and 2.3 times lower than that of the blank, respectively. We confirmed that the standard deviations were relatively low and the results were statistically significant (*P*‐value < .05) of the target gene concentration of 0.01‐10 ng/µL against the blank and nontarget template. The SERS‐based detection of the DNA intercalating dye of our system showed approximately 100‐fold enhancement in sensitivity as compared to conventional fluorescence‐based detection methods. Because the developed detection method starts from high signal intensity and measures signal reduction by the target molecules, so it exhibit higher signal‐to‐noise ratio and can be more sensitive especially for low target concentration than the method that measures generating signal by the target molecules from the zero signal intensity.^26^, ^27^ On the other hand, when the only EVAGreen‐specific SERS signal intensity at 870 cm^−1^ was plotted according to the target gene concentration without correction with the reference dye, the EVAGreen signal was reduced in the presence of target gene compared with the blank and the nontarget template, but it showed high standard deviations, *P*‐value > 0.5 below target gene concentration of 1 ng/µL, and relatively low correlation with the target gene concentration (Figure S2). Therefore, we confirmed that the correction of EVAGreen SERS signal intensity by ROX SERS signal intensity was useful to evaluate the SERS‐DNA detection. The combination of the other EVAGreen SERS peaks and the other ROX SERS peaks showed similar results.

**FIGURE 5:**
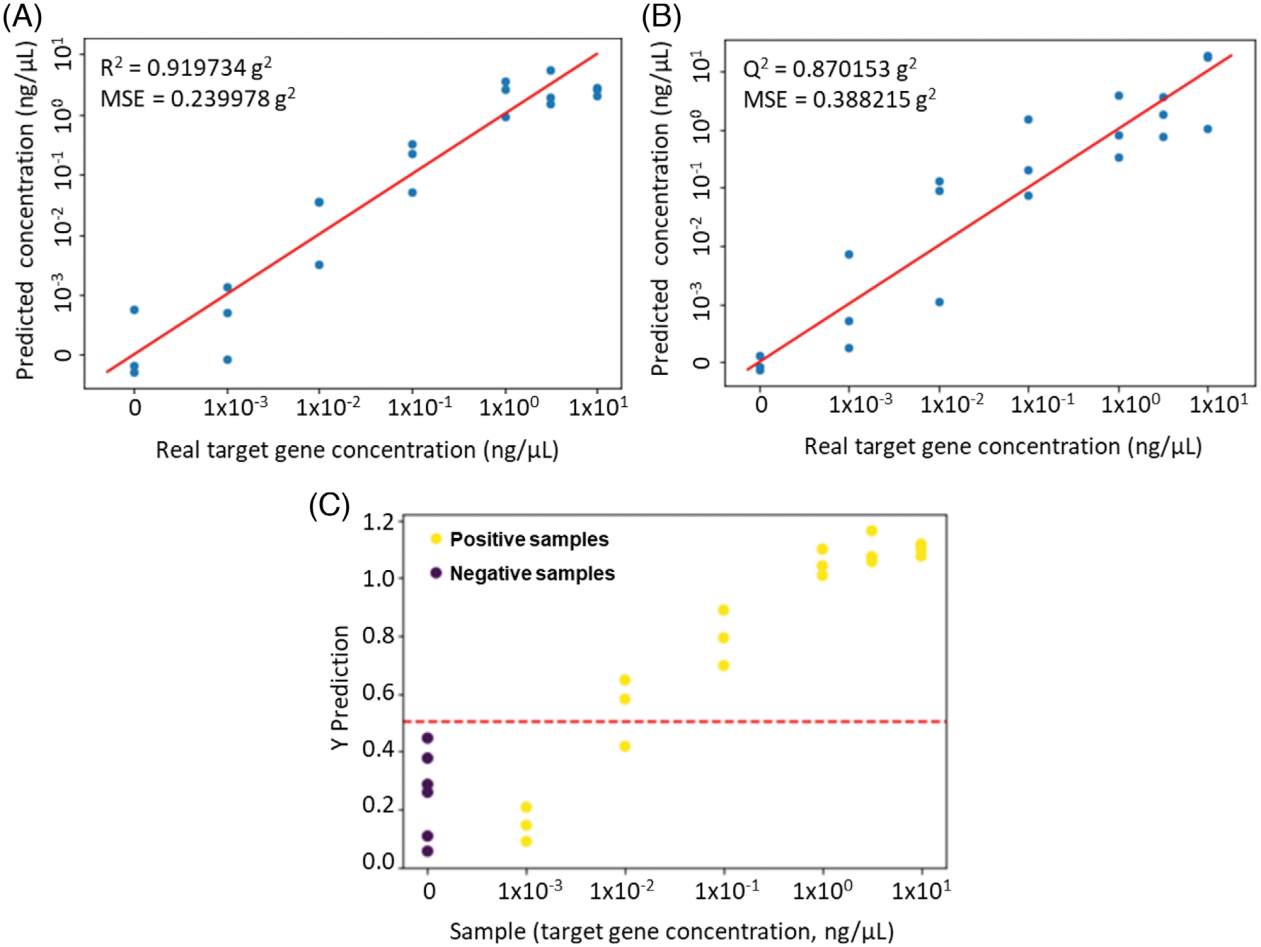
Multivariate analysis of PCR‐SERS detection by PLS regression for (A) training set and (B) test set, and (C) PLS‐DA

For more comprehensive quantitative analysis and validation, we carried out multivariate analysis by PLS regression using all EVAGreen‐specific peaks (615, 870, 1235, 1368, 1490, and 1529 cm^−1^) and all ROX‐specific peaks (692, 755, 840, 1224, 1452, and 1501 cm^−1^). A logarithmic plots of the predicted target gene concentration versus the true target gene concentration for training set and test set are shown in Figure 5A and B. Although the points from blank and the lowest target gene concentration of 0.001 ng/µL were slightly overlapped in the prediction, a strong relationship was observed between the true values and the predicted values by the PLS method (*R* ^2^ = .92 and mean squared error (MSE) = .24 for training set and *Q* ^2 ^= .87 and MSE = .388 for test set). PLS discriminant analysis (PLS‐DA) was further performed for classification of positive samples and negative samples (blank and nontarget template) as shown in Figure 5C. The optimum threshold for sample classification plotted as the dashed line was set to 0.5. On the *Y* or cutoff line, those above the 0.5 point were indicated positive samples and below that point were indicated negative samples. The PLS‐DA showed that except for all samples with the lowest target gene concentration of 0.001 ng/µL and one sample with a target gene concentration of 0.01 ng/µL, all positive samples were clearly distinguished from the negative samples. The result of multivariate analysis by PLS was similar to the quantitative analysis using individual peak intensities, which showed that PCR‐SERS detection improved the sensitivity by 10‐100 times compared to conventional fluorescence‐based detection methods of real‐time PCR and gel electrophoresis.

### 3.5 LAMP‐SERS detection

**FIGURE 6:**
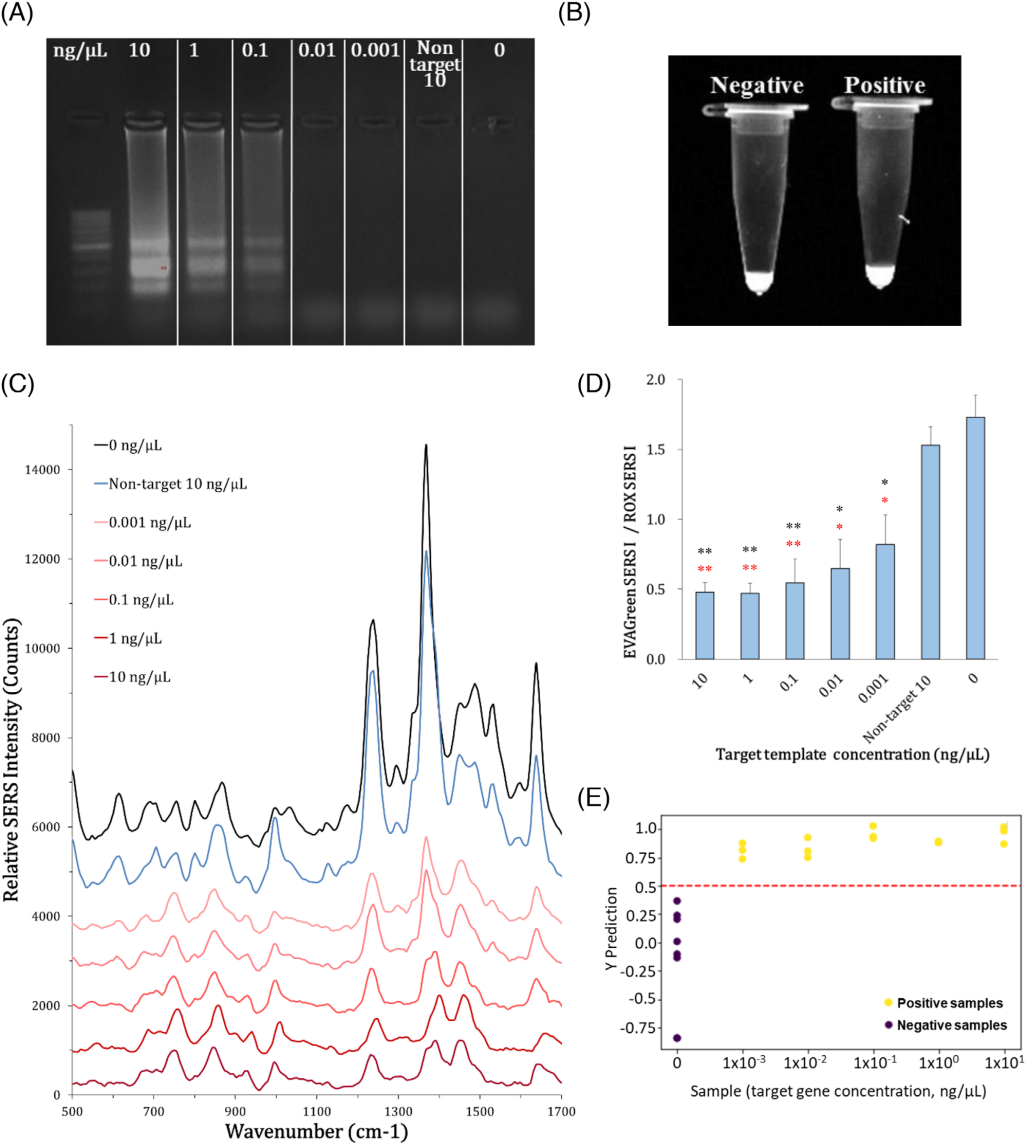
LAMP analysis. (A) Agarose gel electrophoresis of LAMP products. (B) Photo image of LAMP products of negative control and positive sample of target template 10 ng/µL with EVAGreen under UV light. (C) SERS spectra of LAMP products with EVAGreen and ROX on the AgNWs‐GFF substrate. (D) Bar graph of the EVAGreen SERS intensity at 870 cm^–1^/ROX SERS intensity at 840 cm^–1^ according to target template concentration. (**P < .005, *P < .05; black * is against black and red * is against nontarget template)

Unlike PCR, LAMP uses four to six primers and does not amplify by doubling. The LAMP products have cauliflower‐like configurations with multiple loops and have mixture of different amplicon sizes.^28^ For LAMP, we used target templates at a final concentration range of 0.001‐10 ng/µL. Blank (without template) and nontarget template 10 ng/µL were also included. We performed LAMP at a constant temperature of 65°C for 30 min, with gel electrophoresis of LAMP products showing target‐specific amplification at a detection limit of 0.1 ng/µL (Figure 6A). On the other hand, the LAMP products containing EVAGreen (3×) did not show any difference between the blank and target template 10 ng/µL under UV light due to fluorescence generated by nonspecific binding of EVAGreen to the LAMP primers (Figure 6B).

SERS spectra of LAMP products with EVAGreen (3×) and ROX (1×) on the AgNWs‐GFF substrate were obtained using the portable Raman equipment and baseline correction was carried out. The LAMP‐SERS spectra showed similar results to the PCR‐SERS spectra. The SERS signals of EVAGreen and ROX were similarly enhanced in the blank and nontarget template. There were no significant signals in the spectra other than EVAGreen‐ and ROX‐specific peaks. In the presence of the target template, the EVAGreen‐specific SERS signals at 615, 870, 1235, 1368, 1490, and 1529 cm^−1^ decreased gradually according to the concentration of the target template, whereas ROX‐specific SERS signals at 692, 755, 840, and 1452 cm^−1^ showed no distinguishable changes. All groups containing the target gene were highly distinguishable from those of the blank and nontarget template based on the relative intensities of the SERS vibrational bands of 800‐900 and 1450‐1550 cm^−1^ without SERS intensity‐based calculations as in the PCR‐SERS spectra (Figure 6C).

The EVAGreen SERS I/ROX SERS I value for LAMP products was calculated and presented as a bar graph (Figure 6D). The EVAGreen SERS I/ROX SERS I values were 1.73, 0.81, 0.64, 0.55, 0.47, 0.48, and 1.55 for the target templates of 0, 0.001, 0.01, 0.1, 1, 10, and nontarget template 10 ng/µL, respectively. We confirmed that the values decreased as the target templateincreased and all values were statistically significant from the blank and nontarget template. The EVAGreen SERS I/ROX SERS I value of the nontarget template was slightly lower than that of the blank, but it was significantly higher than those of the target template groups. At the target template concentrations of 0.001 and 0.01 ng/µL that were not detectable by the gel electrophoresis, their EVAGreen SERS I/ROX SERS I value was about 2.1 and 2.7 times lower than that of the blank. The results suggested that this approach improved detection sensitivity by approximately 100‐fold as compared with gel electrophoresis.

The PLS regression analysis was also performed for the LAMP‐SERS detection as above PCR‐SERS detection. However, because the amount of LAMP amplicons was generally higher than that of PCR amplicons and the signal was already saturated at the lowest target gene concentration of 0.001 ng/µL, there was no significant correlation in quantification by PLS regression. On the other hand, the PLS‐DA for LAMP‐SERS detection is shown in Figure 6E. In this model, the optimum threshold for sample classification was set to 0.5. All positive samples were clearly distinguished from the negative samples. Taken together, the LAMP‐SERS detection system was able to detect down to a target gene concentration of 0.001 ng/µL, which was 100‐fold‐enhanced sensitivity than that of the conventional fluorescence‐based detection method of gel electrophoresis.

## 4 CONCLUSIONS

We developed a highly sensitive and simple DNA‐SERS detection method using the AgNWs‐GFF substrate. The AgNWs‐GFF substrate was fabricated by vacuum filtration of AgNWs coated with a negatively charged PVP onto GFF. On the substrate, the SERS intensities of EVAGreen intercalated into amplified DNA from PCR and LAMP decreased gradually depending on the target‐template concentration due to large size and/or negative charge of DNA, whereas that of ROX remained nearly constant. With this phenomenon, we were able to detect the presence of the DNA amplicon simply by observing the relative intensities of the SERS vibrational bands. The ROX could be used as an internal standard to correct the SERS intensity associated with EVAGreen to quantify the DNA amplicon without a calibration curve. Furthermore, multivariate analyses by PLS regression and PLS‐DA showed similar results with the quantitative analysis using individual peak intensities. Taken together, the PCR‐SERS and LAMP‐SERS detections showed high repeatability and about 100‐fold sensitivity relative to the conventional fluorescence assays such as real‐time PCR and gel electrophoresis. It is expected that the amplification time for the target gene may be reduced based on the high sensitivity. The DNA‐SERS detection method can be also applied to various isothermal amplification methods such as recombinase polymerase amplification, helicase‐dependent isothermal amplification, strand displacement amplification, and so on. The DNA‐SERS detection method can open a new on‐site molecular diagnostic system based on high sensitivity, repeatability, simplicity, affordability, and convenience.

## Supporting information

Supporting information
