# Supplementary material for: Highly sensitive and repeatable DNA‐SERS detection system using silver nanowires‐glass fiber filter substrate
Source: Anal Sci Adv. 2020 Oct 16;2(7-8):397–407. doi: 10.1002/ansa.202000096 (PMC10989524; doi:10.1002/ansa.202000096)
Supplement: Supplementary file 1 — Supporting information [file ANSA-2-397-s001.docx]

**Supporting information**

**Highly Sensitive and Reproducible DNA-SERS Detection System using Silver Nanowires-Glass Fiber Filter Substrate**

Ayoung Woo,^1^ Kyongmook Lim,^2^ Baek Hwan Cho,^1,2^* Ho Sang Jung,^3^* Min-Young Lee^1,2^*

^1^Department of Medical Device Management and Research, Samsung Advanced Institute for Health Sciences & Technology, Sungkyunkwan University, 81, Irwon-ro, Gangnam-gu, Seoul, 06351, Republic of Korea

^2^ Biomedical Engineering Research Center, Samsung Medical Center, 81, Irwon-ro, Gangnam-gu, Seoul, 06351, Republic of Korea

^3^Advanced Nano-Surface Department, Korea Institute of Materials Science (KIMS), Changwon, Gyeongnam 51508, Republic of Korea


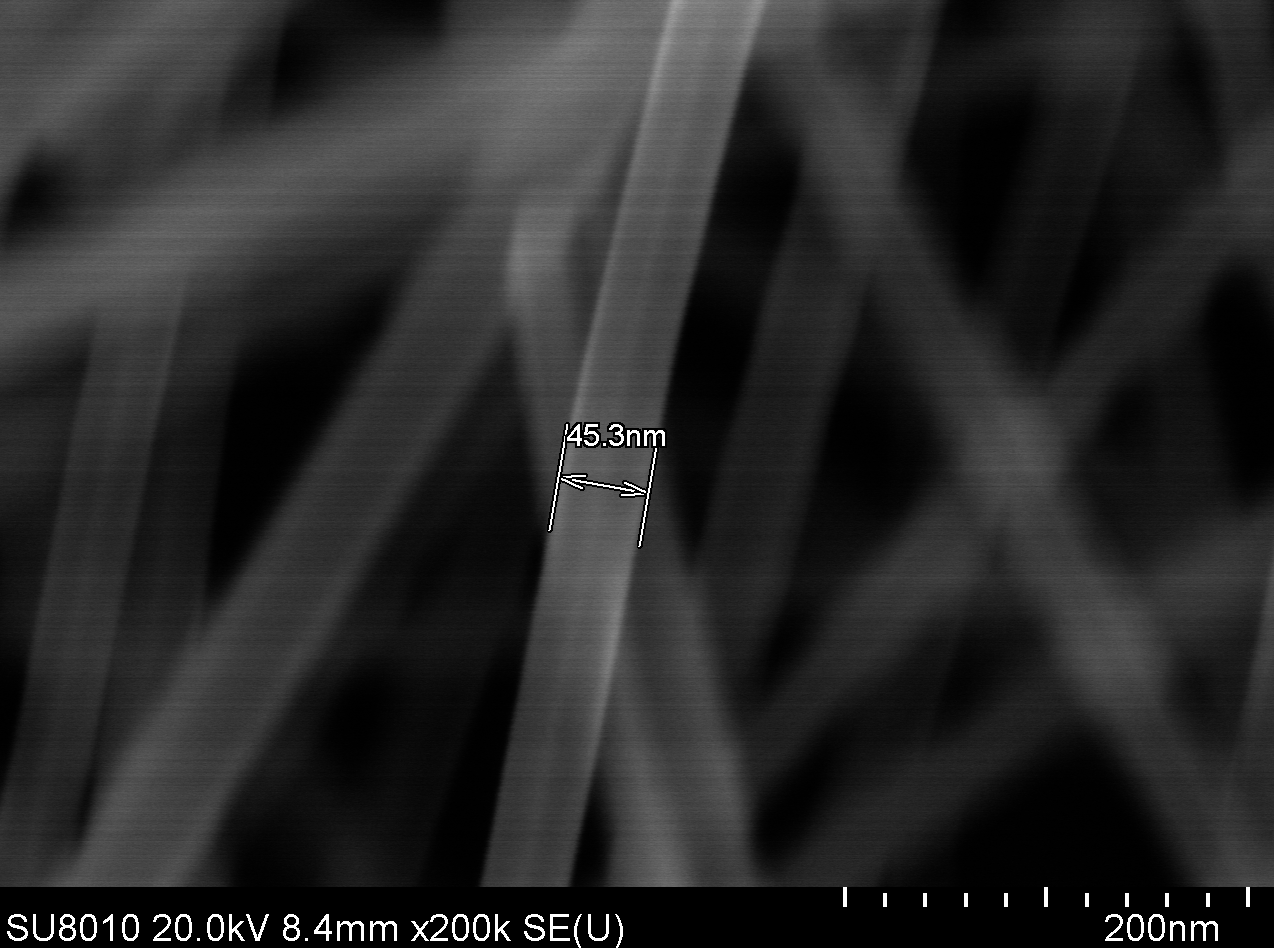


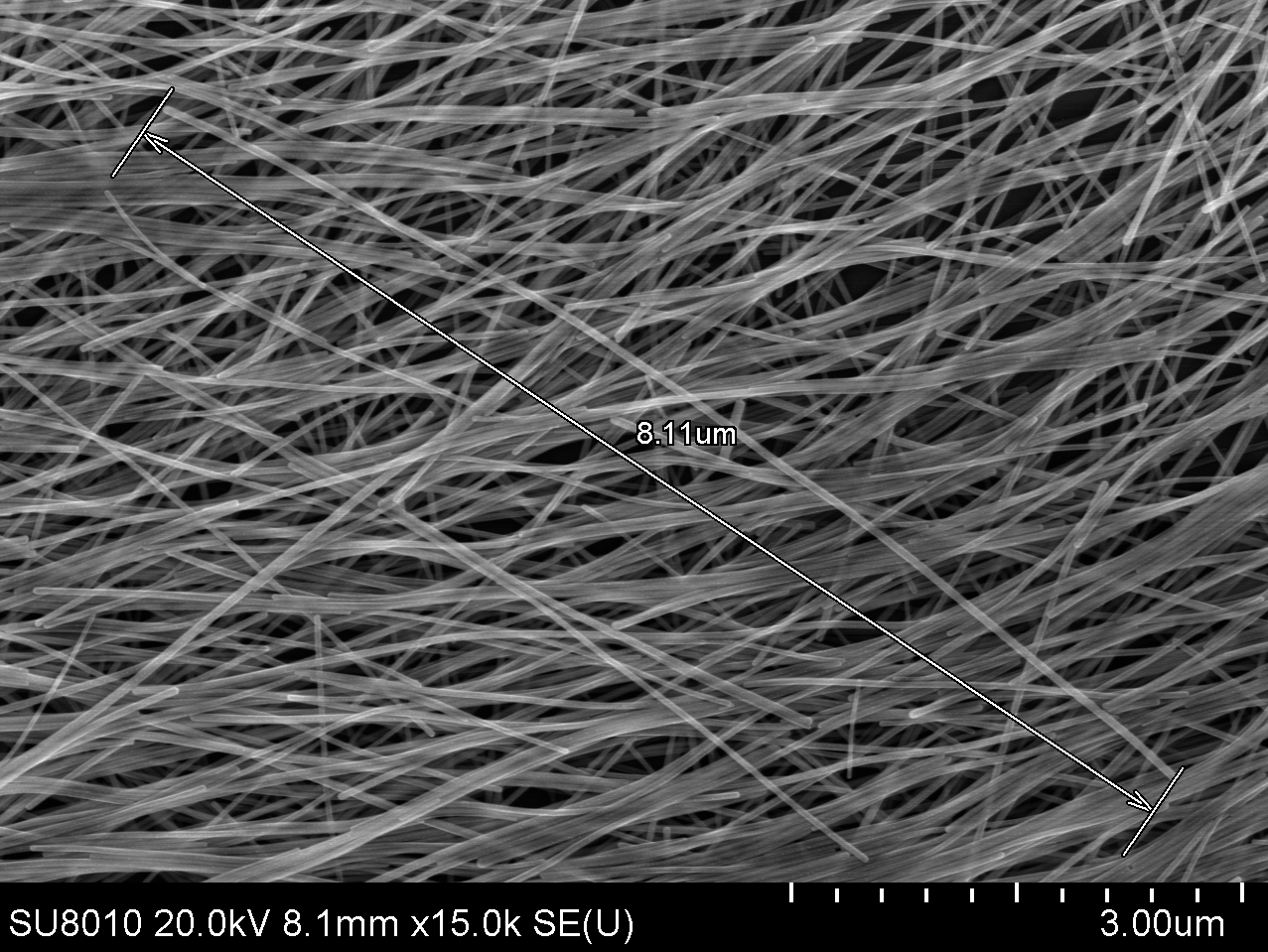


**Figure S1**. SEM images of AgNWs.


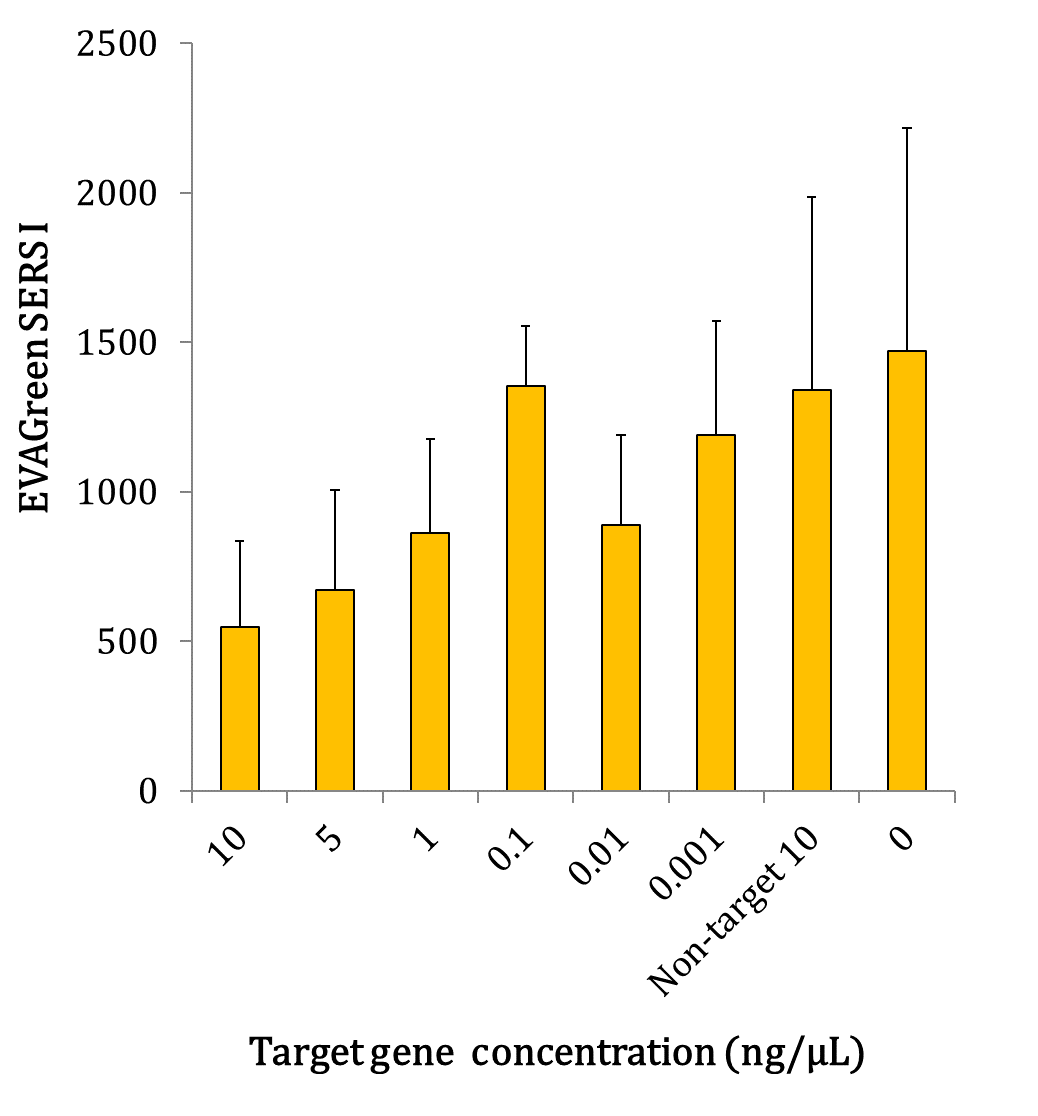


*

*

*

*

*

**Figure S2**. Bar graph of the EVAGreen SERS intensities at 870 cm^-1^ according to target template concentration. * P<0.05; * is against blank and * is against non-target template)
